# Extracellular matrix protein turnover markers are associated with axial spondyloarthritis—a comparison with postpartum women and other non-axial spondyloarthritis controls with or without back pain

**DOI:** 10.1186/s13075-022-02839-1

**Published:** 2022-06-23

**Authors:** Helena Port, Signe Holm Nielsen, Sofie Falkenløve Madsen, Anne-Christine Bay-Jensen, Morten Karsdal, Sengül Seven, Inge Juul Sørensen, Lone Morsel-Carlsen, Mikkel Østergaard, Susanne Juhl Pedersen

**Affiliations:** 1 Nordic Bioscience A/S, Immunoscience, Herlev hovedgade 207, 2730 Herlev, Denmark; 2 University of Copenhagen, Clinical Medicine, Copenhagen, Denmark; 3 Technical University of Denmark, Biomedicine and Biotechnology, Kgs. Lyngby, Denmark; 4 Rigshospitalet, Center for Arthritis Research, Glostrup, Denmark; 5 Frederiksberg Hospital, Radiology, Frederiksberg, Denmark

**Keywords:** Axial spondyloarthritis, Extracellular matrix, Biomarkers

## Abstract

**Background:**

Axial spondyloarthritis (axSpA) is a common chronic inflammatory disease, associated with extracellular matrix (ECM) remodeling of the cartilage, bone, and connective tissues. The primary symptom of axSpA is back pain, caused by inflammation. However, there is a medical need to truly identify patients with axSpA from other subjects with buttock or low back pain attributable to other reasons. We aimed to investigate circulating biomarkers of ECM/inflammation (MMP-degraded type I (C1M), II (C2M, T2CM), III (C3M), IV (C4M), VI (C6M), and X (C10C, COL10NC) collagens, CRPM, PROM and VICM) and ECM formation of type II (PRO-C2), III (PRO-C3), IV (PRO-C4), and VI (PRO-C6) collagens as potential biomarkers to identify patients with axSpA.

**Methods:**

We measured biomarkers from a cross-sectional study with 204 participants by enzyme-linked immunosorbent assay (ELISA). The study included axSpA patients (*N* = 41), women with postpartum buttock/pelvic pain (*N* = 46), disc herniation (*N* = 25), and a group of healthy subjects (including women without postpartum pelvic pain (*N* = 14), subjects with various types of physical strain (cleaning staff (*N* = 26) long-distance runners (*N* = 23)), and healthy men (*N* = 29)). Differences between the groups were calculated by ANCOVA and AUC, while Spearman’s correlations were performed with ECM biomarkers and clinical scores.

**Results:**

Patients with axSpA expressed significantly higher levels of C1M, C4M, and VICM (*p* < 0.05-*p* < 0.0001) compared to all the non-axSpA control groups. Further, C6M and PRO-C4 were significantly higher in patients with axSpA (both *p* < 0.0001) compared to women with postpartum pelvic pain and healthy subjects, whereas PRO-C3 was significantly lower compared to healthy subjects (*p* = 0.01). The best ECM common biomarker to differentiate between axSpA and the non-axSpA control groups was PRO-C4 (AUC ≥ 0.75; specificity ≥ 0.79, sensitivity = 0.65). Mild correlations were observed between collagen turnover and inflammation biomarkers and CRP and MRI (*ρ* ≥ 0.3; *p* < 0.05-*p* < 0.001).

**Conclusions:**

Biomarkers of type I, IV, and VI collagen and biomarkers of inflammation showed an altered turnover in patients with axSpA compared with the non-axSpA control groups. Such biomarkers may be useful in combination with MRI or independently to separate patients with axSpA from other back pain conditions.

**Supplementary Information:**

The online version contains supplementary material available at 10.1186/s13075-022-02839-1.

## Background

Axial spondyloarthritis (axSpA) is a chronic inflammatory rheumatic disease that predominantly affects the axial skeleton. The clinical characteristics defining axSpA are chronic back pain and sacroiliitis displayed on magnetic resonance imaging (MRI), and with time, patients may get progressive ankylosis of the sacroiliac (SI) joint and spine leading to ankylosing spondylitis (AS) [1]. AxSpA generally manifests in early adulthood, between the ages of 20 and 30 years [1], and early diagnosis is important since several effective treatments are available [2, 3]. Diagnosis of the early stages of axSpA is still currently a clinical challenge since chronic low back pain is a common symptom. Also, MRI detected bone marrow edema in the sacroiliac joints is also frequently seen in patients with nonspecific back pain [4, 5] and particularly in woman with postpartum pelvis/buttock pain [6, 7], athletes [6, 8], and among other groups of healthy subjects [7, 9], where MRI may have less diagnostic value. Thus, there is an unmet medical need for improved methods for diagnosis. In axSpA, systemic inflammation is currently measured by C-reactive protein (CRP), which however often is within the normal range in many axSpA patients [10] and, therefore, is not always reflective of disease activity. CRP may also be elevated as a result of various other chronic inflammatory conditions and cannot be characterized as axSpA specific [11]. Thus, more precise biomarkers reflecting axSpA pathology are needed to identify the altered inflammation occurring within the affected joints [12], which also may have clinical utility in diagnosis and monitoring of disease activity.

The extracellular matrix (ECM) is formed by collagens, elastin, laminin, fibronectin, and proteoglycans. Type I, II, III, IV, and VI collagens are the main structural elements of the ECM [13]. Prolargin and vimentin are ECM proteins related to inflammation, which are found in connective tissue and in cartilage and tendon, respectively. ECM proteins are degraded by proteases, such as cysteine proteases, matrix metalloproteases (MMP), and serine proteases, which release protease-specific metabolites [10]. MMP are the most crucial enzymes in the ECM remodeling, which support cell proliferation, differentiation and apoptosis, and participate in the turnover of ECM [14]. It has been observed that ECM remodeling of the cartilage, bone and connective tissues, and subsequent inflammation is disturbed in axSpA [10]. MMP-3, MMP-8, and MMP-9 have been shown to reflect the increased disease activity and structural progression in AS [15]. MMP-3 in particular, is produced in response to cytokines in the joints, and higher MMP-3 serum levels have been found in patients with SpA compared to healthy controls [16].

When proteases degrade the ECM, metabolites are generated and detection of those that are derived in the specific/local tissue can reflect local pathology and be quantified in blood [17]. Metabolites, also called neoepitopes, of type I (C1M), type III (C3M), and type VI (C6M) collagen reflect the degradation of soft tissues, while neoepitopes of type II collagen (C2M, T2CM) reflect degradation of cartilage. C1M has previously shown to be elevated in serum of patients with AS, psoriatic arthritis (PsA), and rheumatoid arthritis (RA) [18, 19]. In two studies, levels of C1M, C2M, C3M, and C4M were significantly elevated in AS patients compared with age-matched controls, indicating that AS patients have higher connective tissue turnover and these biomarkers were associated with disease activity [12, 18]. The neoepitopes of type X collagen (C10C, COL10NC) detect chondrocyte activity [20]. Formation biomarkers of type II, III, IV, and VI collagen (PRO-C2, PRO-C3, PRO-C4, and PRO-C6, respectively) quantify formation of cartilage (PRO-C2), fibrosis (PRO-C3 and PRO-C6), and basement membrane turnover (PRO-C4). In a study performed by Gudmann et al., PRO-C2 and PRO-C10, a marker of type X collagen formation, presented increased levels in serum samples from axSpA and PsA patients compared to healthy subjects [20]. Metabolites generated by degradation of C-reactive protein (CRPM) and prolargin (PROM) reveal inflammation-related processes and the citrullinated vimentin neoepitope (VICM) shows macrophage activity [14, 21]. It has been shown that CRPM is associated with disease activity in axSpA and together with VICM, both biomarkers separated AS from non-radiographic axSpA patients [14, 22, 23]. All together, these ECM-derived metabolites can be quantified in serum but requires further validation before they can be applied as biomarkers to identify patients with axSpA.

In this study, we investigated the circulating ECM biomarkers and their potential to differentiate axSpA patients from subjects with or without buttock or pelvic pain attributed to other reasons, particularly post-partum women, as well as a large group of healthy subjects. In addition, we aimed to investigate the association of ECM-derived biomarkers with clinical parameters and whether these biomarkers could separate axSpA from non-axSpA controls.

## Methods

### Subjects

A total of 204 participants were included in the MASH study—a prospective, cross-sectional study conducted at Rigshospitalet, Copenhagen, Denmark, from 2013 to 2016 [7]. The MASH study investigated the MRI and biochemical markers in patients with axSpA, subjects with back pain of other reasons, subjects with strain on the sacroiliac joints, and healthy subjects. The participants were included according to seven predefined groups: (1) patients with axSpA (*N* = 41), (2) women with postpartum buttock/pelvic pain 4 months to 16 month after delivery (*N* = 46), (3) women without postpartum buttock/pelvic pain 4 months to 16 months after delivery (*N* = 14), (4) patients with lumbar disc herniation (*N* = 25), (5) cleaning staff (*N* = 26), (6) long-distance runners (*N* = 23), (7) a group of healthy men (*N* = 29). Cleaning staff and long-distance runners were included to have a group which had repeated strain in daily life. All participants signed informed consent before study inclusion. The study was approved by the local ethical committee (approval no. H-17034960) and conducted in accordance with the Declaration of Helsinki V and Danish legislation.

Demographic and clinical data were acquired from all participants. Participants were evaluated for exclusion criteria (described in [7]) to ensure that the non-axSpA controls did not have axSpA. Detailed methodology of the clinical examination and the MRI data acquisition of all participants has been previously described [7]. Serum samples for biomarker measurement were collected from all participants and stored at -80 °C until analysis. Missing data from biomarker measurements were observed in the following biomarkers: VICM (4 data points), PROM (1 data point), and C2M (1 data point) due to insufficient sample. The seven groups were reduced to four groups upon biomarker analysis: (1) patients with axSpA, (2) women with postpartum buttock/pelvic pain, (3) patients with lumbar disc herniation, and (4) a new group generated including all healthy subjects which comprised the other four groups: women without postpartum buttock/pelvic pain, cleaning staff, long-distance runners, and healthy men.

### ECM biomarker measurements

A panel of ECM turnover biomarkers was measured in serum using validated ELISAs and IDS automated chemiluminescent assay.

We measured the biomarkers: MMP-degraded type I (C1M) [24], II (C2M [25] and T2CM [26]), III (C3M) [27], IV (C4M) [28], and IV(C6M) [29] collagen, type X (C1OC [30] and COL10NC) collagen degradation, C-reactive protein metabolite (CRPM) [31], MMP-cleaved prolargin (PROM) [32], citrullinated and MMP-degraded vimentin (VICM) [21], and type II (PRO-C2) [33], III (PRO-C3) [34], IV (PRO-C4) [35], and IV (PRO-C6) [36] collagen formation (Table 1). All biomarker analyses were quality controlled with two kit control and three in house quality controls. The inter- and intra- assay variations were below 15% and 10%, respectively. Sample measurements were performed in duplicates and were accepted when the standard curve had a coefficient of variance ≤ 10% and if at least three of the five control samples had a coefficient of variance ≤ 20%.

**Table 1:** Panel of biomarkers used in the study

Biomarker	Description of the biomarker	Implication	Previously assessed in axSpA or AS
C1M	MMP-2/9/13-degraded type I collagen	Interstitial matrix degradation	Yes
C2M	MMP (multiple)-degraded type II collagen	Cartilage degradation	Yes
T2CM	Collagenase-degraded type II collagen	Cartilage degradation	No
C3M	MMP-9-degraded type III collagen	Interstitial matrix degradation	Yes
C4M	MMP (multiple)-degraded type IV collagen	Primarily basal lamina disruption	Yes
C6M	MMP-degraded type VI collagen	Microfibril degradation	Yes
C10C	Cathepsin-K-mediated degradation of type X collagen	Chondrocyte activity	No
COL10NC	NC1 domain of type X collagen	Chondrocyte activity	No
PROM	MMP-cleaved prolargin	Interstitial matrix degradation	No
VICM	Citrullinated and MMP-degraded vimentin	Inflammation	Yes
CRPM	C-reactive protein metabolite	Inflammation	Yes
PRO-C2	Type II collagen N-terminal pro-peptide	Cartilage formation	Yes
PRO-C3	Type III collagen N-terminal pro-peptide	Fibrosis	No
PRO-C4	Type IV 7S domain collagen	Basement membrane formation	No
PRO-C6	Type VI collagen C5 domain	Fibrosis	No

### Statistics

Baseline characteristics are described as number (frequency) and percentage for categorical variables, and as mean (± SD) for continuous variables. Kruskal-Wallis rank test was used to examine baseline differences between axSpA patients and the other groups of participants. The biomarker data was natural log-transformed for normalization and four new ratios of degradation/formation of type II (C2M/PRO-C2), III (C3M/PRO-C3), IV (C4M/PRO-C4), and VI (C6M/PRO-C6) collagen were also included and natural log-transformed. Differences in the biomarker levels and type II, III, IV, and VI collagen degradation/formation ratios among patients with axSpA and each of the other groups were examined by ANCOVA analyses adjusting by confounders. A nominal significance level of 5% was used for the analyses and adjustment for multiple comparison were performed within each biomarker, by Tukey method, but not across biomarkers. Spearman’s correlation test was used in analyses between the biomarker levels and clinical assessments in the patients with axSpA, women with postpartum pain, and patients with disc herniation. Only biomarkers showing one or more correlations with a rho (*ρ*) value ≥ 0.3 or ≤ − 0.3 were included. An area under the receiver operating characteristic curve (AUC) was used for exploring the separation potential of the metabolites among patients with axSpA and non-axSpA independently. Data analyses were performed using R studio version 4. 0. 3 (R Foundation for Statistical Computing, Vienna, Austria. URL https://www.R-project.org; 2020). Graphical illustrations were created using GraphPad Prism version 9.00 for Windows (GraphPad Software, GraphPad Software, San Diego, California USA, www.graphpad.com).

## Results

### Characteristics of participants

Demographic, clinical, and biochemical characteristics of the 204 participants included in the MASH study were stratified according to the group classification and presented in Table 2. Overall, 84 (41.2%) of the participants were male, the mean age was 33.2 years (range 19–45), and 22% were HLA-B27 positive. In addition, 36 (17.6%) of the participants were daily smokers and 70 (34.3%) practiced moderate physical activity. Patients with axSpA exhibited higher inflammatory back pain, Spondyloarthritis Research Consortium of Canada (SPARCC) sacroiliac joint inflammation and structural scores compared to the rest of the groups, and 12 patients had a medical history of arthritis, 6 of enthesitis, 9 of uveitis, 1 of psoriasis, and 4 of inflammatory bowel disease.

**Table 2:** Demographic, clinical, and biochemical characteristics of the different groups of study participants

	Patients with axSpA (*n* = 41)	Women with postpartum buttock/pelvic pain (*n* = 46)	Patients with disc herniation (*n* = 25)	Healthy subjects (*n* = 92)
Demographic feature				
Age, years	30.9 (6.41)	32.6 (3.25 )^†^	35.2 (5.70)^†^	34 (6.42)^†^
Male sex, no. (%)	26 (63.4)	0 (0)^§^	11 (44.0)	45 (48.9)
HLA-B27 positive, no. (%)	33 (80.5)	5 (10.9)^§^	0 (0)^§^	6 (6.5)^§^
CRP, mg/ liter	11.4 (13.5)	1.66 (2.06)^§^	2.19 (3.28)^§^	1.71 (3.01)^§^
Body mass index, kg/m^2^	23.1 (2.95)	25.0 (4.39)	26.1 (4.03)	24.50 (3.45)
No. of childbirths if woman	1.7 (0.8)	1.5 (0.8)	1.6 (0.9)	2.14 (1.13)
Years since last childbirth if woman	4.9 (4.6)	0.7 (0.3)	9.1 (7.0)	7 (6.8)
Symptom duration, years	8 (5.6)	1 (0.8)^§^	0.8 (0.4)^§^	NA
Smoking status, no (%)				
Daily smoker	10 (24.4)	2 (4.3)	12 (48.0)	12 (13)
Occasional smoker	9 (22.0)	6 (13.0)	4 (16.0)	11 (12)
Never smoked	13 (31.7)	25 (54.3)	7 (28.0)	62 (67.4)
Previous smoker	8 (19.5)	13 (28.3)	2 (8.0)	7 (7.6)
Physical activity (%)				
Light	11 (26.8)	7 (15.2)	9 (36.0)	17 (18.5)
Moderate	11 (26.8)	29 (63.0)	7 (28.0)	23 (25)
Heavy	13 (31.7)	8 (17.4)	6 (24.0)	21 (22.8)
Most heavy	6 (14.6)	2 (4.3)	3 (12.0)	31 (33.7)
Inflammatory back pain, no. (%)	41 (100)	11 (23.9)^§^	3 (12.0)^§^	0 (0)
Medical history of SpA features, no (%)				
Arthritis	12 (29.3)	0 (0)^§^	1 (4.0)	0 (0)
Enthesitis	6 (14.6)	1 (2.2)^†^	0 (0)	9 (9.8)
Uveitis	9 (22.0)	0 (0)^§^	0 (0)	0 (0)^§^
Psoriasis	1 (2.4)	0 (0)	0 (0)	0 (0)
Inflammatory bowel disease, no. (%)	4 (9.8)	0 (0)	0 (0)	0 (0)^‡^
Clinical examination				
SJC ≥ 1, no. (%)	1 (2.4%)	NA	NA	NA
TJC ≥ 1, no. (%)	2 (4.8%)	NA	NA	NA
Pain	3.9.8 (25.1)	NA	NA	NA
Physician global VAS score	4.3 (2.7)	NA	NA	NA
BASDAI score	4 (2,1)	NA	NA	NA
BASFI score	2.7 (2.1)	NA	NA	NA
BASMI score	1.2 (1.7)	0.3 (0.7)^§^	0.5 (0.6)	2.85 (7.19)^§^
SPARCC SI joint score				
Inflammation score (0–48)	10.8 (10.7)	3.88 (5.7)^‡^	0.3 (0.9)^§^	0.41 (1.01)^§^
SSS fat lesion score (scale 0–40)	12 (11.1)	0.5 (2.3)^§^	0.3 (1.0)^§^	0.6 (2)^§^
SSS erosion score (scale 0–40)	5.2 (4.8)	0.5 (2.2)^§^	0.02 (0.1)^§^	0.05 (0.2)^§^
SSS backfill score (scale 0–20)	4.8 (5.6)	0 (0)^§^	0 (0)^§^	0 (0)^§^
SSS ankylosis score (scale 0–20)	3.5 (6.0)	0 (0)^§^	0 (0)^§^	0 (0)^§^
Extracellular biomarkers levels, ng/ml				
C1M	84.3 (85.7)	36.7 (20.2)	42.4 (28.1)	34.20 (20.98)
C2M	23.97 (6.98)	28.12 (9.70)	27.65 (19.25)	23.16 (6.60)
T2CM	5.56 (1.35)	5.66 (1.66)	5.96 (1.44)	5.71 (2.60)
C3M	15.6 (3.95)	14.0 (1.90)	13.9 (4.46)	13.84 (3.04)
C4M	34.9 (10.2)	27.5 (4.59)	28.0 (8.64)	28.03 (8.74)^§^
C6M	20.5 (5.75)	17.2 (2.83)	19.4 (4.85)	16.89 (4.52)^§^
C10C	2570 (462)	2610 (450)	2530 (632)	2557.12 (593.15)
COL10NC	9.15 (5.81)	9.33 (13.2)	8.32 (4.95)	9.78 (5.32)
PROM	0.30 (0.09)	0.26 (0.09)	0.27 (0.08)	0.26 (0.08)
VICM	5.80 (4.36)	3.85 (2.63)^‡^	3.65 (3.09)	3.65 (2.38)
CRPM	11.9 (2.87)	11.0 (5.16)^‡^	12.9 (12.4)	10.42 (2.55)^‡^
PRO-C2	22.39 (6.27)	25.47 (10.30)	21.88 (6.91)	27.10 (20.93)
PRO-C3	10.2 (2.52)	11.2 (2.87)	11.2 (3.70)	11.40 (2.79)^‡^
PRO-C4	7370.07 (763.99)	6811.70 (687.48)^§^	7083.46 (1149.44)	6491.39 (834.36)^§^
PRO-C6	6.94 (2.45)	7.93 (3.51)	7.61 (2.87)	6.11 (1.36)

Except where indicated otherwise, mean ± SD is presented. Kruskal-Wallis rank test was used with patients with patients with axSpA as the reference group. Significance is shown as ^†^*p*< 0.05, ^‡^*p*< 0.01, and ^§^*p*< 0.001

*Abbreviations*: *CRP* C-reactive protein, *NA* not applicable, *SJC* swollen joint count, *TJC* tender joint count, *SPARCC* Spondyloarthritis Research Consortium of Canada, *VAS* visual analog scale (each scale 0–10), *BASDAI* Bath Ankylosing Spondylitis Disease Activity Index (scale 0–10), *BASFI* Bath Ankylosing Spondylitis Functional Index (scale 0–10), *BASMI* Bath Ankylosing Spondylitis Metrology Index (scale 0–10), *SSS* SI joint structural lesion score, *C1M* metalloproteinase (MMP)-degraded type I collagen, *C2M* metalloproteinase (MMP)-degraded type II collagen, *T2CM* MMP-1 and MMP-13-mediated degradation of type II collagen, *C3M* MMP-degraded type III collagen, *C4M* MMP-degraded type IV collagen, *C6M* MMP-degraded type VI collagen, *CRPM* C-reactive protein metabolite, *PROM* MMP-1 and MMP-13-mediated degradation of prolargin, *VICM* citrullinated and MMP-degraded vimentin, *PRO-C2* pro-peptide of type II collagen, *PRO-C3* pro-peptide of type III collagen, *PRO-C4* pro-peptide of type IV collagen, *PRO-C6* pro-peptide of type VI collagen

### Demographic description

#### ECM turnover is enhanced in patients with axSpA compared to women with pelvic postpartum pain, patients with disc herniation, and healthy controls

Patients with axSpA expressed significantly higher levels of MMP-degraded type I, IV, and VI collagen (C1M, C4M and C6M; *p* < 0.0001, *p* < 0.05, *p* < 0.0001, respectively), MMP-driven citrullinated vimentin degradation (VICM, *p* < 0.05), type IV collagen formation (PRO-C4, *p* < 0.0001), and degradation/formation ratio of type VI collagen (C6M/PRO-C6, *p* < 0.0001) compared to women with buttock/pelvic postpartum pain when adjusting for confounders age, gender and body mass index (BMI) (Table 3). Patients with axSpA also presented significantly higher levels of C1M, C4M, VICM, and degradation/formation ratio of type III collagen (C3M/PRO-C3; *p* < 0.05, *p* ≤ 0.01, *p* ≤ 0.01, *p* < 0.05, respectively) when compared to patients with disc herniation (Table 3). Furthermore, when compared to healthy controls, patients with axSpA showed significantly higher levels of the same biomarkers as when compared to women with pelvic postpartum pain (C1M, C4M, C6M, VICM, PRO-C4, C3M/PRO-C3; *p* < 0.0001, *p* < 0.0001, *p* < 0.0001, *p* < 0.01, *p* < 0.0001, *p* < 0.01, respectively) and significant decreased levels of type III collagen formation (PRO-C3, *p* ≤ 0.01). Similar significant differences were observed when further adjusting for the participants’ physical activity (from light to most heavy) or their smoking status (from daily smoker to never smoked) (Table 3). C-reactive protein metabolite (CRPM) and type III collagen degradation (C3M) levels were higher in patients with axSpA compared to healthy subjects when further adjusting for their smoking status (*p* < 0.05 in both) and type IV degradation/formation ratio (C4M/PRO-C4) was significantly lower in patients with axSpA compared to patients with disc herniation (*p* < 0.05). However, when further adjusting for MRI inflammation score, no significant differences were found in C4M or VICM among patients with axSpA vs. women with pelvic postpartum pain, vs. patients with disc herniation or vs. healthy controls (Table 3). No significant differences were observed in biomarkers of type II and VI collagen formation (PRO-C2 and PRO-C6, respectively), type II collagen degradation (C2M and T2CM) or type II degradation/formation ratio (C2M/PRO-C2), type X collagen degradation (C10C and COL10NC), nor MMP-cleaved prolargin (PROM) among the three comparisons in any of the adjusted models (Table 3).

**Table 3:** Comparison of serum-tested biomarkers levels between groups

	Model A: Adjusted for age, gender, and BMI			Model B: Adjusted for age, gender, BMI, and physical activity			Model C: Adjusted for age, gender, BMI, and smoking status			Model D: Adjusted for age, gender, BMIMMP and SPARCC MRI SI joint inflammation score		
	AxSpA vs WWPP	AxSpA vs DH	AxSpA vs HS	AxSpA vs WWPP	AxSpA vs DH	AxSpA vs HS	AxSpA vs WWPP	AxSpA vs DH	AxSpA vs HS	AxSpA vs WWPP	AxSpA vs DH	AxSpA vs HS
C1M	**< 0.0001**	**0.04**	**< 0.0001**	**< 0.0001**	0.05	**< 0.0001**	**0.002**	**0.03**	**< 0.0001**	**0.02**	0.54	**0.01**
C2M	0.67	0.95	0.78	0.77	0.93	0.88	0.71	0.87	0.94	0.74	0.98	0.82
T2CM	1.00	0.96	0.93	1.00	0.96	0.89	0.98	0.86	0.73	0.99	1.00	0.64
C3M	0.59	0.14	0.11	0.62	0.18	0.32	0.55	0.07	**0.03**	1.00	0.91	0.99
C4M	**0.02**	**0.01**	**< 0.0001**	**0.02**	**0.02**	**< 0.0001**	**0.03**	**0.01**	**< 0.0001**	0.18	0.28	0.09
C6M	**< 0.0001**	0.38	**< 0.0001**	**0.001**	0.44	**< 0.0001**	**0.002**	0.29	**< 0.0001**	**0.01**	0.78	**0.01**
C10C	0.90	0.75	0.79	0.89	0.73	0.73	0.78	0.81	0.71	0.72	0.48	0.48
COL10NC	1.00	0.99	0.66	1.00	0.99	0.76	1.00	0.97	0.93	0.97	0.74	1.00
PROM	0.66	0.94	0.11	0.82	0.94	0.16	0.62	0.95	0.11	0.63	0.90	0.17
VICM	**0.04**	**0.01**	**0.002**	**0.05**	**0.01**	**0.003**	0.11	**0.002**	**0.002**	0.22	0.16	0.17
CRPM	0.32	0.84	0.10	0.74	0.91	0.54	0.20	0.85	**0.04**	0.53	0.98	0.43
PRO-C2	0.59	1.00	0.50	0.93	1.00	0.85	0.71	1.00	0.61	0.76	0.99	0.82
PRO-C3	0.44	0.36	**0.01**	0.44	0.38	**0.02**	0.79	0.26	0.07	0.47	0.42	**0.05**
PRO-C4	**< 0.0001**	0.17	**< 0.0001**	**< 0.0001**	0.20	**0.000**	**0.002**	0.20	**< 0.0001**	**0.01**	0.60	**< 0.0001**
PRO-C6	0.20	0.40	0.54	0.46	0.43	0.31	0.35	0.43	0.21	0.16	0.34	0.88
C2M/PRO-C2	0.99	0.98	0.32	1.00	0.96	0.68	1.00	0.97	0.54	1.00	0.96	0.61
C3M/PRO-C3	0.22	**0.04**	**0.002**	0.23	**0.05**	**0.01**	0.39	**0.01**	**0.002**	0.67	0.43	0.20
C4M/PRO-C4	0.51	0.08	0.24	0.50	0.09	0.33	0.55	0.04	0.14	0.89	0.49	0.92
C6M/PRO-C6	**< 0.0001**	0.07	0.15	**< 0.0001**	0.08	0.52	**< 0.0001**	0.06	0.44	**< 0.0001**	0.15	0.37

ANCOVA analysis between groups were performed. Comparisons that had statistical significance of *p* < 0.05 are highlighted in bold

*Abbreviations*: *WWPP* women with pelvic postpartum pain, *DH* disc herniation, *HS* healthy subjects, *C1M* metalloproteinase (MMP)-degraded type I collagen, *C2M* metalloproteinase (MMP)-degraded type II collagen, *T2CM* MMP-1 and MMP-13-mediated degradation of type II collagen, *C3M* MMP-degraded type III collagen, *C4M* MMP-degraded type IV collagen, *C6M* MMP-degraded type VI collagen, *PROM* MMP-1 and MMP-13-mediated degradation of prolargin, *VICM* citrullinated and MMP-degraded vimentin, *CRPM* C-reactive protein metabolite, *C10C* cathepsin-K mediated degradation of type X collagen, *COL10NC* NC domain of type X collagen, *PRO-C2* pro-peptide of type II collagen, *PRO-C3*pro-peptide of type III collagen, *PRO-C4* pro-peptide of type IV collagen, *PRO-C6* pro-peptide of type VI collage

We also explored for differences between patients with axSpA and each of the initial seven groups (Supplementary Figure S1). Similar differences were observed within the same biomarkers as described above. C1M, C3M, C4M C6M, CRPM, PRO-C4, and VICM showed significantly higher levels in patients with axSpA than in the runners’ group (*p* < 0.01 for all except VICM (*p* < 0.05)), and the C3M/PRO-C3 ratio was also significantly increased (*p* < 0.001). C1M, C6M, PRO-C4, and VICM showed significantly increased levels in the axSpA group compared to the healthy men group. PRO-C4 also showed higher levels in the axSpA group compared to the cleaning staff group and the women with pelvic postpartum pain group (*p* < 0.001, *p* < 0.01, respectively). C6M/PRO-C6 ratio showed significantly higher levels in the group of patients with axSpA compared to the women with pelvic postpartum pain (*p* < 0.01). Neither C2M, T2CM, PRO-C2, C2M/PRO-C2, C4M/PRO-C4, nor PROM showed any significant differences between the axSpA group and each of the other groups; see Figure S1.

#### ECM remodeling biomarkers are not strongly associated with clinical assessment of disease activity or severity of axSpA

In patients with axSpA, PRO-C3 and C3M/PRO-C3 mild–moderately correlated with age (Spearman’s *ρ* ≥ ±0.35, *p* < 0.05-*p* < 0.001, Table 4). C1M, C3M, C4M, C6M, PRO-C4, and C3M/PRO-C3 moderate–highly correlated with CRP (*ρ* ≥ 0,5; all *p* < 0.001, Table 4), while COL10NC, VICM, CRPM, and C6M/PRO-C6 presented a mild-moderate correlation (*ρ* ≥ ±0.35; *p* < 0.05-*p* < 0.01, Table 4). C3M, PRO-C3, and C3M/PRO-C3 mild–moderately correlated with symptom duration of the disease (*ρ* > ±0.34; *p* < 0.05-*p* < 0.001, Table 4). Pain visual analog scale (VAS) mildly correlated with PRO-C6 and C6M/PRO-C6 (*ρ* ≥ ±0.35; *p* < 0.05, Table 4). Bath Ankylosing Spondylitis Disease Activity Index (BASDAI) presented a mild correlation with PRO-C3 (*ρ* = − 0.31; *p* < 0.01), whereas Bath Ankylosing Spondylitis Metrology Index (BASMI) showed a mild to moderate correlation with C6M and C6M/PRO-C6 (*ρ* ≥ 0.37; *p* < 0.05 and *p* < 0.001, Table 4). The SPARCC MRI SI joint inflammation score was mildly correlated with C10C (*ρ* = − 0.32; *p* < 0.05). From the MRI SI Joint Structural Scores (SSS), erosion presented a mild correlation with C10C, and C6M/PRO-C6 (*ρ* ≥ ±0.3; *p* < 0.01) and a moderate to high correlation with PRO-C6 (*ρ* = 0.55; *p* < 0.001). SSS Backfill score presented a mild to moderate correlation with T2CM, C10C, and PRO-C4 (all *ρ* > ±0.32; *p* < 0.05), whereas SSS ankylosis score was mild-moderately correlated with C3M, C4M, C6M, PROM, C3M/PRO-C3, C4M/PRO-C4, and C6M/PRO-C6 (all *ρ* > ±0.34; *p* < 0.05-*p* < 0.01).

**Table 4:** Correlations of the biomarkers with clinical variables in patients with axSpA

	Age	Body mass index	CRP	Symptom duration	Pain VAS	Global VAS	BASDAI	BASFI	BASMI	SPARCC MRI SI joint inflammation score (0–48)	SSS fat lesion score (scale 0–40)	SSS erosion score (scale 0–40)	SSS backfill score (scale 0–20)	SSS ankylosis score (scale 0–20)
C1M	− 0.04	− 0.19	**0.93**^**§**^	0.20	0.20	0.12	0.12	0.23	0.28	0.23	0.00	0.07	0.17	0.25
C2M	0.04	− 0.05	0.18	0.08	0.04	0.04	0.04	0.23	0.12	− 0.21	0.00	− 0.28	0.12	0.06
T2CM	0.09	− 0.05	− 0.08	0.21	− 0.13	− 0.07	0.03	0.11	0.06	− 0.17	0.03	0.05	**0.35**^**†**^	0.09
C3M	0.06	− 0.12	**0.56**^**§**^	**0.34**^**†**^	0.10	0.05	0.04	0.08	0.05	0.26	0.14	0.21	0.21	**0.42**^**‡**^
C4M	0.03	− 0.11	**0.51**^**§**^	0.15	0.16	0.10	0.03	− 0.06	0.08	0.19	0.24	0.10	0.14	**0.43**^**‡**^
C6M	0.05	− 0.04	**0.72**^**§**^	0.29	0.07	0.13	0.03	0.21	**0.40**^**‡**^	0.09	0.04	− 0.04	0.13	**0.35**^**†**^
C10C	0.25	− 0.14	− 0.18	0.16	0.01	0.01	0.07	0.07	− 0.10	**− 0.32**^**†**^	− 0.09	− 0**.30**^**†**^	− 0**.36**^**†**^	− 0.29
COL10NC	0.17	0.02	**− 0.35**^**†**^	0.06	− 0.09	0.06	0.13	0.02	0.19	− 0.22	− 0.06	− 0.15	0.00	0.06
PROM	− 0.05	− 0.21	0.17	0.06	0.18	0.16	0.07	0.24	0.22	− 0.11	0.18	− 0.10	0.16	**0.34**^**†**^
VICM	0.21	− 0.03	**0.47**^**‡**^	0.15	0.17	0.17	0.07	0.17	0.03	0.20	− 0.11	0.07	0.09	0.25
CRPM	− 0.14	− 0.26	**0.44**^**‡**^	− 0.03	0.12	− 0.01	− 0.12	0.06	0.14	0.11	0.15	− 0.11	− 0.07	0.26
PRO-C2	− 0.08	− 0.13	0.13	0.19	− 0.19	− 0.06	− 0.06	− 0.13	0.12	− 0.04	− 0.01	0.16	0.10	0.07
PRO-C3	**− 0.54֛**^**§**^	0.14	− 0.21	**− 0.51**^**§**^	− 0.26	− 0.25	**− 0.31**^**†**^	− 0.28	− 0.20	0.18	− 0.09	0.20	− 0.10	− 0.29
PRO-C4	− 0.19	− 0.03	**0.63**^**§**^	0.11	0.14	0.08	0.08	0.18	0.19	0.18	0.08	0.23	**0.32**^**†**^	0.23
PRO-C6	− 0.28	0.03	0.11	0.09	**− 0.36**^**†**^	− 0.23	− 0.25	− 0.08	− 0.04	0.22	− 0.21	**0.55**^**§**^	0.07	− 0.18
C2M/PRO-C2	0.08	0.07	0.14	− 0.01	0.15	0.10	0.09	0.30	0.03	− 0.06	0.05	− 0.21	0.08	0.08
C3M/PRO-C3	**0.35**^**†**^	− 0.16	**0.55**^**§**^	**0.53**^**§**^	0.26	0.22	0.24	0.23	0.14	0.13	0.20	0.08	0.22	**0.47**^**‡**^
C4M/PRO-C4	0.07	− 0.13	0.28	0.11	0.11	0.07	− 0.04	− 0.14	0.01	0.14	0.17	0.05	0.00	**0.34**^**†**^
C6M/PRO-C6	0.26	− 0.02	**0.49**^**‡**^	0.14	**0.35**^**†**^	0.25	0.21	0.22	**0.37**^**†**^	− 0.06	0.12	**− 0.39**^**†**^	0.04	**0.37**^**†**^

Spearman’s correlation between serological metabolites and clinical scores were performed. Spearman’s rho (*ρ*) is shown. The bold *ρ* determine the most relevant correlations (*ρ* > 0.3 and *ρ* < − 0.3). Significance of correlations are shown as ^†^*p*< 0.05, ^‡^*p*< 0.01, and ^§^*p*< 0.001

*Abbreviations*: *C1M* metalloproteinase (MMP)-degraded type I collagen, *C3M* MMP-degraded type III collagen, *C4M* MMP-degraded type IV collagen, *C6M* MMP-degraded type VI collagen, *PRO-C3* pro-peptide of type III collagen, *PRO-C4* pro-peptide of type IV collagen, *VICM* citrullinated and MMP-degraded vimentin, *CRPM* C-reactive protein metabolite, *SPARCC* Spondyloarthritis Research Consortium of Canada, *BASDAI* Bath Ankylosing Spondylitis Disease Activity Index (scale 0–10), *BASFI* Bath Ankylosing Spondylitis Functional Index (scale 0–10), *BASMI* Bath Ankylosing Spondylitis Metrology Index (scale 0–10), *SSS* SI joint structural score, *SJC* swollen joint count, *TJC* tender joint count

C1M, T2CM C3M, C4M, C6M, COL10NC, PROM, VICM, CRPM, PRO-C3, PRO-C4, PRO-C6 C3M/PRO-C3, C4M/PRO-C4, and C6M/PRO-C6 were not correlated with BMI, VAS patient global, BASDAI, BASFI, swollen joint count (SJC), tender joint count (TJC), SPARCC SSS fat score, and SSS erosion score (Table 4). C2M, PRO-C2, and C2M/PRO-C2 did not show any correlation with any of the clinical variables.

We further examined the correlation between ECM metabolites and clinical parameters in the women with pelvic postpartum pain and in the patients with disc herniation (Table S1, Table S2). From the clinical variables, we could include age, BMI, CRP, symptom duration, BASMI, and SPARCC SI joint inflammation score. In women with postpartum pain, C6M, PRO-C4, and the ratio C4M/PRO-C4 presented a mild correlation with BMI (*ρ* ≥ ±0.33; *p* < 0.05-*p* < 0.01). C4M, C6M, and PROM were mildly correlated with CRP (*ρ* ≥ 0.31; *p* < 0.05), whereas PRO-C4 presented a moderate correlation (*ρ* = 0.47, *p* < 0.01). COL10NC, PRO-C6, CRPM, and the ratio C6M/PRO-C6 presented a mild-moderate correlation with symptom duration (*ρ* ≥ ±0.33; *p* < 0.05-*p* < 0.01). C10C, PRO-C4, and C4M/PRO-C4 were mild–moderately correlated with BASMI (*ρ* ≥ ±0.3; *p* < 0.05-*p* < 0.01), and only C3M presented a mild correlation with inflammation score (*ρ*> 0.3; *p* < 0.05). C3M, C6M, PRO-C4, PRO-C6, CRPM, C10C, COL10NC, C4M/PRO-C4, and C6M/PRO-C6 did not show any relevant correlation with age (Table S1). C1M, C2M, T2CM, VICM, PRO-C2, PRO-C3, C2M/PRO-C2, and C3M/PRO-C3 did not present any relevant correlation with the clinical parameters.

In patients with disc herniation, C4M presented a moderate correlation with age (*ρ* = 0.47; *p* < 0.001), whereas CRPM and the ratio C4M/PRO-C4 presented a mild correlation (*ρ* ≥ 0.31; *p* < 0.001). CRPM also presented a mild correlation with BMI (*ρ* = 0.37; *p* < 0.05). C1M and PROM showed a mild correlation with CRP (*ρ* ≥ ±0.33, *p* < 0.01), whereas PRO-C4 and C3M/PRO-C3 showed a moderate correlation (*ρ* ≥ ±045, *p* < 0.01-*p* < 0.001). PRO-C4 mildly correlated with symptom duration (*ρ* = − 0.32; *p* < 0.05), whereas PRO-C6 showed a moderate correlation (*ρ* = − 0.58; *p* < 0.05). C2M mildly correlated with BASMI (*ρ* > 0.31; all *p* < 0.01), and together with PRO-C3, it showed a mild to moderate correlation with SPARCC SI joint inflammation score (*ρ* ≥ − 0.35; *p* < 0.05-*p* < 0.01). C1M, C2M, T2CM, COL10NC, VICM, PRO-C2, C2M/PRO-C2, and C6M/PRO-C6 did not present any relevant correlation with the clinical parameters.

#### Diagnostic utility of biomarker levels for axial spondyloarthritis

CRP presented the highest AUC in axSpA vs. women with postpartum pain (AUC = 0.80, Table 5). CRP was also the best metabolite for the identification of axSpA from disc herniation patients and healthy controls. The AUC from axSpA vs. disc herniation patients was 0.71 and vs. healthy controls was 0.83 (Table 5). Moreover, for identifying axSpA from women with pelvic postpartum pain, inflammation biomarkers VICM and PROM as well as PRO-C4 had higher AUC than the rest of tested biomarkers (Table 5). To discriminate between axSpA and healthy subjects, PRO-C4, C6M, and C1M presented the second, third, and fourth highest AUC after CRP. However, the rest of biomarkers presented weak AUCs for differentiating patients with axSpA from women with pelvic postpartum pain, patients with disc herniation and control subjects (AUC < 0.73, Table 5).

**Table 5:** Diagnostic value of biomarker levels for axSpA

	AxSpA vs women with postpartum pain				AxSpA vs disc herniation patients				AxSpA vs healthy controls			
	AUROC [95% CI]	T	S	SP	AUROC [95% CI]	T	S	SP	AUROC [95% CI]	T	S	SP
**C1M**	**0.66 [0.50–0.82]**	66.2	0.96	0.43	0.59 [0.40–0.78]	67.8	0.92	0.43	**0.73 [0.61–0.86]**	72.7	0.98	0.43
**C2M**	0.41 [0.24–0.58]	18.2	0.25	0.87	0.38 [0.18–0.58]	35.2	1.00	0.043	0.56 [0.42–0.70]	19.8	0.44	0.78
**T2CM**	0.60 [0.43–0.77]	5.4	0.71	0.61	0.51 [0.30–0.72]	5.6	0.75	0.48	0.56 [0.42–0.70]	5.4	0.65	0.61
**C3M**	0.50 [0.32–0.68]	18.1	1.00	0.26	0.45 [0.25–0.66]	15.9	0.75	0.35	0.54 [0.39–0.69]	20.1	0.98	0.22
**C4M**	0.65 [0.47–0.82]	33.9	0.92	0.57	0.57 [0.35–0.78]	28.1	0.67	0.61	**0.68 [0.54–0.82]**	33.7	0.81	0.57
**C6M**	**0.68 [0.51–0.84]**	19.6	0.88	0.57	0.47 [0.27–0.68]	23.1	0.75	0.35	**0.73 [0.59−0.87]**	19.4	0.90	0.57
**C10C**	0.42 [0.25–0.59]	2924.8	0.88	0.22	0.49 [0.28–0.70]	1698.7	0.083	1.00	0.61 [0.47–0.75]	2332	0.54	0.70
**COL10NC**	0.45 [0.28–0.62]	13.7	0.96	0.22	0.50 [0.29–0.72]	13.5	0.92	0.22	0.36 [0.21–0.51]	16.4	0.96	0.13
**PROM**	**0.75 [0.61−0.89]**	0.27	0.83	0.65	0.59 [0.40–0.79]	0.34	0.92	0.43	**0.69 [0.56−0.82]**	0.34	0.91	0.43
**VICM**	**0.77 [0.63−0.91]**	3.6	0.79	0.70	0.68 [0.48–0.89]	2.6	0.58	0.83	**0.72 [0.58−0.85]**	3.0	0.59	0.78
**CRPM**	0.65 [0.49–0.81]	11.1	0.75	0.57	0.58 [0.37–0.80]	11.5	0.75	0.52	0.62 [0.47–0.77]	11.7	0.75	0.52
**PRO-C2**	0.43 [0.26–0.60]	21.6	0.50	0.57	0.54 [0.31–0.78]	16.7	0.42	0.91	0.47 [0.33–0.61]	16.7	0.15	0.91
**PRO-C3**	0.48 [0.31–0.65]	10.8	0.67	0.43	0.39 [0.20–0.58]	11.5	0.75	0.3	0.36 [0.21–0.50]	18.1	0.98	0.043
**PRO-C4**	**0.75 [0.61−0.89]**	7227	0.79	0.65	0.59 [0.39–0.80]	7575.1	0.83	0.43	**0.82 [0.73−0.92]**	7224.1	0.88	0.65
**PRO-C6**	0.39 [0.22–0.55]	10.1	0.92	0.13	0.45 [0.23–0.66]	12.5	1.00	0.087	0.60 [0.45–0.74]	5.1	0.31	0.91
**C2M/PRO-C2**	0.46 [0.28–0.63]	0.54	0.12	1.00	0.43 [0.20–0.66]	0.91	0.42	0.74	0.56 [0.41–0.70]	1.1	0.65	0.57
**C3M/PRO-C3**	0.53 [0.36–0.71]	2.2	1.00	0.22	0.49 [0.28–0.70]	2.3	1.00	0.22	0.61 [0.46–0.75]	1.2	0.48	0.74
**C4M/PRO-C4**	0.53 [0.35–0.72]	0.004	0.75	0.52	0.56 [0.36–0.77]	0.0052	0.92	0.35	0.53 [0.36–0.69]	0.005	0.85	0.39
**C6M/PRO-C6**	**0.71 [0.56**–0**.86]**	3.0	0.79	0.61	0.56 [0.35–0.77]	2.5	0.42	0.78	0.61 [0.47–0.76]	3.1	0.73	0.57
**CRP**	**0.80 [0.67–0.93]**	1.9	0.88	0.61	**0.71 [0.54–0.88]**	2.5	0.83	0.57	**0.83 [0.73–0.93]**	1.8	0.85	0.65

The analyses were performed using AUROC, and threshold, sensitivity and specificity are provided. AUC that had statistical significance of *p* < 0.001 are highlighted in bold

*Abbreviations*: *AUROC* area under receiver operator characteristics curve, *T* threshold (ng/ml), *S* sensitivity, *SP* specificity, *CI* confidence interval, *C1M* metalloproteinase (MMP)-degraded type I collagen, *C2M* metalloproteinase (MMP)-degraded type II collagen, *T2CM* MMP-1 and MMP-13-mediated degradation of type II collagen, *C3M* MMP-degraded type III collagen, *C4M* MMP-degraded type IV collagen, *C6M* MMP-degraded type VI collagen, *C10C* cathepsin-K-mediated degradation of type X collagen, *COL10NC* NC domain of type X collagen, *PROM* MMP-1 and MMP-13-mediated degradation of prolargin, *VICM* citrullinated and MMP-degraded vimentin, *CRPM* C-reactive protein metabolite, *CRP* C reactive protein, *PRO-C2* pro-peptide of type II collagen, *PRO-C3* pro-peptide of type III collagen, *PRO-C4* pro-peptide of type IV collagen, *PRO-C6* pro-peptide of type VI collagen

## Discussion

The present study evaluated the ability of ECM turnover biomarkers to separate patients with axSpA particularly from women with postpartum pelvic pain 4 to 16 months after delivery, but also patients with disc herniation, and healthy subjects, the latter including women without postpartum pelvic pain, subjects with various types of physical strain (i.e., cleaning staff and long-distance runners), and healthy men. The data suggest an increased rate of type I, IV, and VI collagen degradation (C1M, C4M and C6M), type III and IV collagen formation (PRO-C3 and PRO-C4), and chronic inflammation (VICM) in axSpA patients compared to women with postpartum pelvic pain, patients with disc herniation, and healthy subjects. The biomarkers C1M, C3M, C4M, C6M, PRO-C4, and CRPM moderate–strongly correlated with CRP. The AUC analysis showed that the overall best ECM biomarker to separate patients with axSpA from both women with postpartum pain and healthy subjects was PRO-C4. To our knowledge, this is the first study to use ECM turnover biomarkers to identify axSpA patients from women with postpartum pelvis pain and other non-axSpA subjects suffering from similar symptoms, and this is important because MRI detected bone marrow edema (BME) in the SI joints is frequent in these groups, where one or more ECM biomarkers potentially may have a clinical utility. These results demonstrate that collagen turnover and inflammation biomarkers derived from the ECM are upregulated in serum from axSpA patients and may indicate disease activity. Such biomarkers may have a future role as diagnostic tools for axSpA together with MRI or potentially independently. In addition, these ECM biomarkers may be useful to separate patients with axSpA from other back pain conditions that challenge diagnostics in routine care.

Recently, early diagnosis has been improved due to advances of MRI techniques; however, it is costly, not widely available, and requires experienced professionals to interpret the findings [3]. Moreover, BME and fat lesions detected by MRI that often are present in patients with axSpA are also frequently seen in non-axSpA subjects, particularly in women with postpartum buttock/pelvic pain [7]. Therefore, a panel of serological biomarkers may aid the need of diagnostic tools to differentiate between patients with axSpA from non-axSpA controls.

We explored a panel of biomarkers to identify the best ECM biomarker for differentiation of patients with axSpA from subjects suffering from other conditions with low back pain or healthy subjects with various types of strain. The levels of C1M, C4M, and C6M were higher in axSpA patients compared to women with postpartum pelvic pain, patients with disc herniation and healthy controls, indicating an enhanced ECM turnover of soft tissue and joint structures. Specifically, C1M and C6M indicate a turnover of the connective tissue, whereas C4M suggests the remodeling of the basal lamina [17]. These results are in agreement with previous findings [12, 18], where serum levels of C1M, C3M, C4M, and C6M were higher in patients with axSpA compared to healthy controls. C2M and T2CM, which are measures of cartilage loss, could not separate axSpA patients from the rest of the non-axSpA groups. In contrast, Bay-Jensen et al. [18] observed higher levels of C2M in patients with AS compared to controls, but the patients in our study had an average symptom duration of 8 years which might differ from the previous study and C2M may be increased in early stages of the disease. Neither type X collagen degradation quantified by C10C nor COL10NC measuring endochondral bone formation was able to differentiate axSpA patients from non-axSpA controls. These findings confirm previously published data, where no differences were found between patients with axSpA and control subjects, albeit they included patients with PsA [20]. Previous findings suggest that chondrocyte hypertrophy may not be the main pathway involved in joint remodeling and fusion in axSpA [37]. Overall, the increased degradation of tissue specific collagens may result in the deficient formation of the structures of the joints, entheses, and adjacent structures and contribute in their biomechanical failure [12]. Other biomarkers reflecting bone metabolism, such as MMP3, have been associated with symptoms and signs of inflammation and structural damage progression and may therefore be a reliable biomarker in axSpA. MMP3 has also shown potential as being a reproducible, sensitive, and specific biomarker reflecting disease activity and associated with MRI measures and clinical assessment parameters [38–41].

Remodeling of the ECM involves the formation of new collagens in the interstitial connective tissue matrix and the basement membrane [13]. We found that levels of type III collagen formation biomarkers were decreased in patients with axSpA compared to the healthy controls, whereas levels of type IV collagen formation biomarker were increased compared to the women with postpartum pelvic pain and healthy controls. This suggest that the excessive turnover of the ECM is not only explained by an increased degradation of collagens, as the formation of collagens is also decreased or increased, which leads to an unbalanced collagen formation/degradation. It has been demonstrated that higher levels of PRO-C3 and PRO-C4 are associated with liver fibrosis [34, 35], whereas PRO-C6 is related with type I diabetes [36]. PRO-C3 but not PRO-C6 is also elevated in patients with RA [42]. According to our results, these biomarkers are also suggested to be relevant in axSpA. We did not find any difference in the levels of type II collagen formation biomarker among the different groups. Luo et al. [33] demonstrated that PRO-C2 was significantly higher in control subjects compared to patients with osteoarthritis, suggesting that a decompensated formation of type II collagen is related to the pathology events.

Chronic inflammation is one of the hallmarks of axSpA [1]. The biomarkers CRPM, PROM, and VICM reflect systemic inflammation, turnover in cartilage, and macrophage activity, respectively [14, 22, 32]. Previously, CRPM has shown to be associated with BASDAI and could segregate AS from nr-axSpA [14], while VICM was associated with burden of AS disease [22]. This is the first study investigating PROM in axSpA, but it has previously been shown to be upregulated in PsA patients [32]. Of the three inflammation biomarkers, we only found that VICM levels were increased in patients with axSpA compared to the non-axSpA groups. Further investigation is needed to study the potential of CRPM, VICM, and PROM as candidates to identify patients with axSpA. Interestingly, the levels of C4M and VICM were no longer significantly different between patients with axSpA and non-axSpA controls, when adjusting for the SPARCC inflammation score, and some of the other biomarkers, such as C1M, C6M, and PRO-C4 were less significant compared to when the other models were used. This may suggest that biomarkers C1M, C6M, and PRO-C4 are dependent on inflammation, reflected by swollen joint BME.

In the current study, clinical data such as symptom duration, clinical indices, and MRI of the SI joints were available and allowed evaluation of ECM turnover biomarkers with disease activity and MRI measurements of inflammation and structural damage. No strong correlations were found between the ECM turnover biomarkers and the clinical data or MRI findings in patients with axSpA. However, C3M and PRO-C3 showed a mild correlation with symptom duration of the disease in patients with axSpA, and C3M presented a mild-moderate correlation with the total SPARCC inflammation score in women with pelvic postpartum pain.

The SPARCC SSS erosion score presented a mild to moderate correlation with C10C and PRO-C6, the backfill score a mild to moderate correlation with T2CM, C10C, and PRO-C4, and the ankylosis score a mild to moderate correlation with the ECM turnover biomarkers C3M, C4M, C6M, and PROM in patients with axSpA. Erosion, backfill, and ankylosis scores reflect bone destruction, early bone formation, and late bone formation, respectively [4]. Our results may suggest that certain biomarkers are better at diagnosing the different stages of axSpA. In agreement with our findings, Bay-Jensen et al. [18] showed a correlation between C3M serum levels and disease activity and structural damage characterized by modified Stoke Ankylosing Spondylitis Spinal Score in AS patients.

CRP presented the best diagnostic capacity to differentiate patients with axSpA from women with pelvic postpartum pain, patients with disc herniation, and healthy controls, while PRO-C4 was the best ECM turnover biomarker for segregating patients with axSpA from women with pelvic postpartum pain and healthy controls. In contrast, Hušáková et al. [12] and Bay-Jensen et al .[18] showed that C3M was the best ECM turnover biomarker for separating AS from asymptomatic controls with an AUC of 0.95 and 0.85, respectively. Moreover, C1M and C4M performed better in segregating AS from asymptomatic controls than CRP (0.90 and 0.96 compared to 0.82), [12]. For true diagnostic purposes, a biomarker panel should probably be generated to guarantee a valid diagnosis and recognition of patients with axSpA from other back pain conditions. Further validation of the biomarkers is needed to confirm their use as diagnostic and more accessible tools than MRI.

The major strength of this study was the heterogeneity in the non-axSpA control group, including subjects with and without back pain. Moreover, all the clinical data regarding the MRI of SI joints obtained from all the subjects of the study allowed us to evaluate correlations between the biomarkers data and local inflammation. Limitations of the study include the sample size and an uneven gender distribution between the groups. Another limitation is the lack of erythrocyte sedimentation rate (ESR), since this has been shown to reflect inflammation in patients with axSpA and would be interesting to compare with the ECM biomarkers [43].

In conclusion, this study showed an altered ECM turnover quantified by biomarkers of type I, III, IV, and VI collagen and inflammation in patients with axSpA compared to non-axSpA subjects. Some associations between the biomarkers and clinical data and MRI scores, respectively, were found. PRO-C4 was the best ECM biomarker for separating patients with axSpA from non-axSpA subjects, but further studies are needed to identify biomarkers as true diagnostic tools. Overall, the investigated biomarkers may potentially be useful, either in combination with MRI or independently, to separate patients with axSpA from other back pain conditions; however, further validations are required.

## Supplementary Information

**Additional file 1: Supplementary Figure S1. **Biomarker levels compared between groups. **Table S1.** Correlations of the biomarkers with clinical variables in women with pelvic postpartum pain. **Table S2.** Correlations of the biomarkers with clinical variables in patients with disc herniation.

## Data Availability

The datasets used and/or analyzed during the current study are available from the corresponding author on reasonable request.

## Abbreviations

AS: Ankylosis spondylitis
AUROC: Area under the receiver operating characteristic curve
AxSpA: Axial spondyloarthritis
BASDAI: Bath Ankylosing Spondylitis Disease Activity Index
BASMI: Bath Ankylosing Spondylitis Metrology Index
BME: Bone marrow edema
C10C: Cathepsin-K-mediated degradation of type X collagen
C1M: MMP-2/9/13-degraded type I collagen
C2M: MMP (multiple)-degraded type II collagen
C3M: MMP-9-degraded type III collagen
C4M: MMP (multiple)-degraded type IV collagen
C6M: MMP-degraded type VI collagen
COL10NC: NC1 domain of type X collagen
CRP: C-reactive protein
CRPM: C-reactive protein metabolite
ECM: Extracellular matrix
MMP: Matrix metalloprotease
MRI: Magnetic resonance imaging
PRO-C2: Type II collagen N-terminal pro-peptide
PRO-C3: Type III collagen N-terminal pro-peptide
PRO-C4: Type IV 7S domain collagen
PRO-C6: Type VI collagen C5 domain
PROM: MMP-cleaved prolargin
PsA: Psoriatic arthritis
RA: Rheumatoid arthritis
SI: Sacroiliac
SPARCC: Spondyloarthritis Research Consortium of Canada
SSS: SI Joint Structural Scores
T2CM: Collagenase-degraded type II collagen
VICM: Citrullinated and MMP-degraded vimentin
