# Supplementary material for: Extracellular matrix protein turnover markers are associated with axial spondyloarthritis—a comparison with postpartum women and other non-axial spondyloarthritis controls with or without back pain
Source: Arthritis Res Ther. 2022 Jun 23;24:152. doi: 10.1186/s13075-022-02839-1 (PMC9219155; doi:10.1186/s13075-022-02839-1)
Supplement: Supplementary file 1 — Additional file 1: Supplementary Figure S1. Biomarker levels compared between groups. Table S1. Correlations of the biomarkers with clinical variables in women with pelvic postpartum pain. Table S2. Correlations of the biomarkers with clinical variables in patients with disc herniation. [file 13075_2022_2839_MOESM1_ESM.docx]

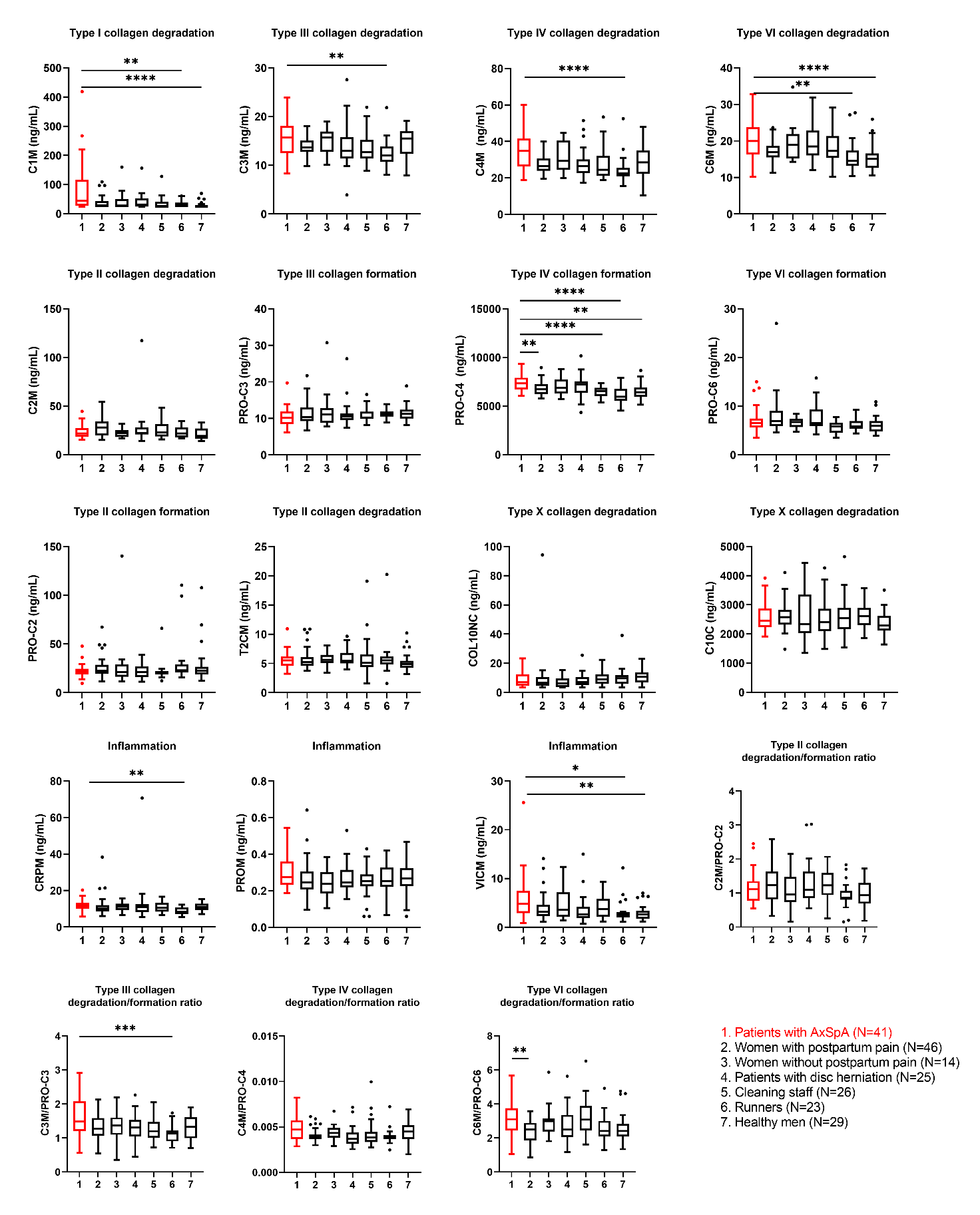
**Figure S1.** Biomarker levels compared between groups.

Linear regression model with pairwise comparisons adjusted by age, gender and BMI was used for differences between patients with axSpA and the rest of the groups. Statistical differences between groups are reported. Asterisks indicate the level of significance, *P<0.05, **P<0.01, ***p<0.001, ****P<0.0001. Data are shown as Tukey´s box plots.

**Table S1.** Correlations of the biomarkers with clinical variables in women with pelvic postpartum pain.

| Biomarker | Age | BMI | CRP | Symptom duration | BASMI | Inflammation score (0-48) |
| --- | --- | --- | --- | --- | --- | --- |
| C1M | 0.20 | 0.10 | 0.24 | 0.16 | -0.11 | -0.06 |
| C2M | 0.27 | -0.09 | -0.10 | -0.11 | 0.21 | 0.10 |
| T2CM | -0.07 | 0.24 | 0.11 | -0.12 | 0.11 | -0.05 |
| C3M | 0.03 | 0.00 | 0.09 | 0.06 | -0.03 | **0.30†** |
| C4M | -0.17 | 0.00 | **0.31†** | 0.11 | -0.01 | 0.24 |
| C6M | 0.02 | **0.33†** | **0.32†** | -0.04 | 0.03 | 0.16 |
| C10C | 0.11 | -0.03 | -0.03 | -0.05 | **-0.39‡** | -0.04 |
| COL10NC | 0.27 | -0.19 | 0.01 | **0.38†** | -0.05 | 0.05 |
| PROM | -0.18 | 0.01 | **0.33†** | -0.01 | -0.13 | 0.08 |
| VICM | -0.10 | 0.12 | 0.07 | -0.01 | 0.04 | -0.15 |
| CRPM | -0.05 | 0.09 | 0.15 | **0.33†** | 0.07 | 0.29 |
| PRO-C2 | -0.09 | 0.22 | 0.24 | 0.08 | 0.11 | 0.00 |
| PRO-C3 | -0.07 | 0.06 | -0.14 | -0.19 | -0.17 | -0.14 |
| PRO-C4 | -0.03 | **0.44‡** | **0.47‡** | -0.05 | **0.39‡** | 0.09 |
| PRO-C6 | -0.07 | 0.06 | 0.10 | **-0.42‡** | -0.13 | -0.18 |
| C2M/PRO-C2 | 0.22 | -0.15 | -0.20 | -0.23 | 0.15 | 0.12 |
| C3M/PRO-C3 | 0.12 | -0.02 | 0.19 | 0.18 | 0.14 | 0.25 |
| C4M/PRO-C4 | -0.11 | **-0.34†** | -0.03 | 0.10 | **-0.30†** | 0.24 |
| C6M/PRO-C6 | 0.07 | 0.10 | 0.07 | **0.38†** | 0.12 | 0.25 |

Spearman´s correlation between serological metabolites and clinical scores were performed Spearman´s rho (ρ) is shown. The bold ρ determine the most relevant correlations (ρ>0.3 and ρ<-0.3). Significance of correlations are shown as † P< 0.05, ‡ P< 0.01 and § P< 0.001. Abbreviations: BASMI: Bath Ankylosing Spondylitis Metrology Index (scale 0–10).

**Table S2.** Correlations of the biomarkers with clinical variables in patients with disc herniation.

| Biomarker | Age | BMI | CRP | Symptom duration | BASMI | Inflammation score (0-48) |
| --- | --- | --- | --- | --- | --- | --- |
| C1M | 0.14 | -0.04 | **0.35‡** | 0.11 | 0.16 | 0.12 |
| C2M | 0.11 | 0.27 | -0.02 | **0.44** | **0.31‡** | **-0.35†** |
| T2CM | 0.24 | -0.10 | 0.20 | -0.28 | -0.05 | -0.06 |
| C3M | 0.27 | 0.05 | 0.29 | -0.15 | 0.14 | -0.13 |
| C4M | **0.47§** | -0.29 | 0.17 | -0.03 | 0.02 | -0.04 |
| C6M | -0.02 | **0.32** | 0.26 | **-0.34** | 0.18 | -0.11 |
| C10C | 0.15 | -0.15 | 0.04 | 0.10 | **0.31** | -0.36 |
| COL10NC | 0.07 | -0.23 | -0.28 | 0.00 | 0.11 | 0.08 |
| PROM | 0.04 | -0.03 | **-0.33†** | -0.18 | -0.15 | -0.17 |
| VICM | -0.29 | -0.09 | -0.18 | -0.20 | -0.04 | -0.04 |
| CRPM | **0.31§** | **-0.37†** | -0.03 | 0.14 | -0.16 | -0.22 |
| PRO-C2 | -0.21 | 0.01 | 0.14 | -0.19 | -0.02 | -0.19 |
| PRO-C3 | 0.21 | 0.09 | -0.37 | 0.16 | 0.01 | **-0.46‡** |
| PRO-C4 | 0.26 | -0.10 | **0.51§** | **-0.58†** | -0.12 | 0.12 |
| PRO-C6 | 0.09 | 0.25 | 0.06 | **-0.32†** | 0.27 | -0.01 |
| C2M/PRO-C2 | 0.19 | 0.09 | -0.23 | 0.21 | 0.20 | -0.02 |
| C3M/PRO-C3 | 0.13 | -0.06 | **0.45‡** | -0.24 | 0.07 | 0.07 |
| C4M/PRO-C4 | **0.39§** | -0.25 | -0.09 | 0.15 | 0.14 | -0.12 |
| C6M/PRO-C6 | -0.08 | -0.03 | 0.18 | 0.19 | 0.06 | -0.04 |

Spearman´s correlation between serological metabolites and clinical scores were performed Spearman´s rho (ρ) is shown. The bold ρ determine the most relevant correlations (ρ>0.3 and ρ<-0.3). Significance of correlations are shown as † P< 0.05, ‡ P< 0.01 and § P< 0.001. Abbreviations: CRP: C-reactive protein; BASMI: Bath Ankylosing Spondylitis Metrology Index (scale 0–10).
